# Prevalence and exposure variables of latent infection by mycobacterium tuberculosis in healthcare workers

**DOI:** 10.1590/0034-7167-2024-0052

**Published:** 2025-01-10

**Authors:** Raphael Sampaio dos Santos, Katerine Moraes dos Santos, Angela Maria Mendes Abreu, Cristiane Cardoso de Paula, Regina Célia Gollner Zeitoune

**Affiliations:** I Universidade Federal do Rio de Janeiro. Rio de Janeiro, Rio de Janeiro, Brazil; II Universidade Federal Fluminense. Rio de Janeiro, Rio de Janeiro, Brazil; III Universidade Federal de Santa Maria. Santa Maria, Rio Grande do Sul, Brazil

**Keywords:** Health Personnel, Latent Tuberculosis, Occupational Health, Tuberculosis, Prevalence, Personal de Salud, Tuberculosis Latent, Salud Laboral, Tuberculosis, Prevalencia

## Abstract

**Objectives:**

To identify in the scientific literature the prevalence, diagnostic methods, and exposure variables of latent infection by *Mycobacterium tuberculosis* in healthcare workers.

**Methods:**

An integrative review of the scientific literature based on the following review question: What are the available scientific evidence in the literature that address the prevalence of latent infection by Mycobacterium tuberculosis in healthcare workers and its association with possible risk factors among these workers?

**Results:**

Being a physician or nurse, being older, and being male were generally associated with higher prevalences. The study also showed that interferon-gamma release assays were more commonly used as a diagnostic method compared to skin tests.

**Conclusions:**

More studies are needed regarding the epidemiology of latent infection by Mycobacterium tuberculosis in the context of healthcare workers, aiming for higher impact actions that contribute to the reduction of tuberculosis worldwide.

## RESUMEN

**Objetivos:**

Identificar en la literatura científica la prevalencia, los medios de diagnóstico y las variables de exposición de la Infección Latente por *Mycobacterium tuberculosis* en trabajadores de la salud.

**Métodos:**

Revisión integrativa de la literatura científica a partir de la siguiente pregunta de revisión: ¿Cuáles son las evidencias científicas disponibles en la literatura que abordan la prevalencia de la infección latente por Mycobacterium tuberculosis en trabajadores de la salud y su asociación con posibles factores de riesgo entre estos trabajadores?

**Resultados:**

Ser médico o enfermero, tener mayor edad y ser del sexo masculino, en general, está asociado a mayores prevalencias. El estudio también mostró que los ensayos de liberación de interferón gamma fueron más utilizados como medio diagnóstico en comparación con las pruebas cutáneas.

**Consideraciones Finales:**

Son necesarios más estudios en relación con la epidemiología de la Infección Latente por Mycobacterium tuberculosis en el contexto de los trabajadores de la salud, con el objetivo de implementar acciones de mayor impacto que contribuyan a la reducción de la Tuberculosis en el mundo.

## INTRODUCTION

Tuberculosis (TB) remains a significant public health problem, with an estimated one-quarter of the global population having been infected with Mycobacterium tuberculosis (MTB). Additionally, the disease has a considerable economic impact on the healthcare services of countries, with affected families facing catastrophic costs (total annual costs suffered by families, related directly and indirectly to TB, exceeding 20% of their income) ^(1)^.

A study conducted in Brazil, which aimed to evaluate the household economic impact of TB illness, revealed that 41% of participating TB patients experienced catastrophic costs. The determining factors for this included being a patient and head of the family, living in poverty before TB, current informal employment, and stopping work during the illness ^(2)^. In this regard, efforts to eliminate TB globally have intensified ^(1)^.

In the context of TB, Latent Infection by Mycobacterium tuberculosis (LTBI) is noteworthy. It is characterized by a persistent immune response to the bacillus without evidence of active disease, but it is of great epidemiological importance because an infected person can develop the disease and transmit it to other healthy individuals, thus sustaining the transmission chain ^(3)^.

Brazil, among other countries, has been striving to achieve the Sustainable Development Goals (SDGs) established by the 2030 Agenda, legitimized in 2015. Hence, this integrative review includes the discussion of LTBI in healthcare professionals, highlighting SDG 3, which aims to ensure health and well-being for all, with one of the targets being the elimination of TB and other epidemics by 2030 ^(4)^.

Healthcare professionals, due to frequent exposure, are part of the at-risk population for acquiring TB and its resistant strains. The risk of disease transmission can vary from one healthcare facility to another, particularly in hospital settings, and even within different areas of the same facility, according to the level of patient circulation^(5)^. Therefore, given the vulnerability of healthcare professionals to LTBI, the risk of progression to active disease, and understanding their role in the TB transmission chain in healthcare settings, it is necessary to comprehend the screening of latent infection by MTB among these workers.

Considering the elimination of TB by 2030 as one of the sustainable development goals and as a global health policy^(4)^, and given the importance of understanding the role of healthcare professionals in the disease transmission chain, this research sought to identify the available scientific evidence in the literature addressing latent infection by MTB in healthcare workers and its association with possible risk factors.

## OBJECTIVE

To identify studies in the scientific literature that address the prevalence of LTBI in healthcare workers and possible exposure variables to TB infection.

## METHODS

### Ethical Aspects

This study used public domain data and did not involve human subjects; therefore, there was no need for review by the Research Ethics Committee.

### Study Design

An integrative review was conducted following methodological procedures: definition of the review question, which helps delimit the topic of interest using the PICo acronym for Population, Interest, and Context elements; search and selection of primary studies, establishing inclusion and exclusion criteria, searching databases, organizing the reference database, and selecting studies; data extraction from the studies using a recording instrument and organizing the extracted data from selected studies; critical evaluation of studies based on the presented results and evidence level; synthesis of the review results and discussion of the evidence, identification of knowledge gaps on the topic, review limitations, and recommendations; presentation of the review ^(6)^.

### Study Period and Location

The search was conducted in May 2022 in the Latin American and Caribbean Center on Health Sciences Information database, accessed through the Virtual Health Library - BVS (BIREME), and the Medical Literature Analysis and Retrieval System Online (MEDLINE), accessed through the National Library of Medicine (PubMed).

### Inclusion and Exclusion Criteria

The inclusion criteria were complete articles directly from the database in Portuguese, English, Spanish, or French that addressed the prevalence of latent infection by MTB, developed in hospital, outpatient, and primary healthcare services, with a population consisting of healthcare workers, regardless of their area of training and level of education. The time frame was the last five years, chosen due to the publication in 2018 of the Protocol for Surveillance of Latent Infection by MTB in Brazil by the Department of Surveillance of Transmissible Diseases. The main objective of this protocol is to provide healthcare professionals and TB control programs with resources for implementing, expanding, and strengthening LTBI surveillance in their territories, thus representing a national political milestone on the topic. Manuals, guidelines, dissertations, theses, guides, editorials, and commentaries were excluded from the analysis.

### Study Protocol

The following review question was formulated: “What scientific evidence is available in the literature addressing the prevalence of latent infection by MTB in healthcare workers and its association with possible risk factors among these workers?” According to the PICo acronym, the population comprised healthcare workers, the phenomenon of interest was latent infection by MTB, and the context was healthcare services. However, inserting related descriptors yielded very unspecific results, so the context was adjusted to occupational health.

To assist in this stage, a Conceptual Map of the PICo strategy was created using the controlled vocabulary of Health Sciences Descriptors (DECS) and Medical Subject Headings (MESH), including alternative terms and entry terms. Search keys were constructed using the controlled vocabulary, employing the Boolean operators OR for integration and AND for differentiation between terms, as shown in Chart 1. Additionally, the PubMed filter for age (above 19 years) was applied, as the focus was on healthcare workers.

**Chart 1:** Search keys used in the databases and the number of results found

Databases	Search Keys	Results
Virtual Health Library (BVS) Using alternative terms	*(Pessoal da Saúde) OR (Prestadores de Cuidados de Saúde) OR (Profissionais da Saúde ) OR (Profissionais de Saúde ) OR (Profissional da Saúde ) OR (Profissional de Saúde ) OR (Trabalhador da Saúde ) OR (Trabalhador de Saúde ) OR (Trabalhadores da Saúde ) OR (Trabalhadores de Saúde) AND (Tuberculose Latente) OR (Infecção Tuberculosa Latente) AND (Saúde do Trabalhador) OR (Higiene do Trabalho) OR (Higiene Industrial) OR (Saúde dos Empregados) OR (Saúde dos Trabalhadores) OR (Saúde Industrial) OR (Saúde Ocupacional) OR (Segurança do Trabalho) OR (Segurança dos Trabalhadores) OR (Segurança no Trabalho) OR (Segurança Ocupacional)*	101
National Library of Medicine (PubMed) Using entry terms	(((((((((((((((((((((((((((((((((((((((((((((((((((((((((((((((((((Health Personnel[Title/Abstract]) OR (Health Personnel[MeSH Terms])) OR (personnel, health[MeSH Terms])) OR (Personnel, Health[Title/Abstract])) OR (Health Care Providers[Title/Abstract])) OR (Health Care Providers[MeSH Terms])) OR (Health Care Provider[MeSH Terms])) OR (Health Care Provider[Title/Abstract])) OR (Provider, Health Care[Title/Abstract])) OR (Provider, Health Care[MeSH Terms])) OR (Healthcare Providers[MeSH Terms])) OR (Healthcare Providers[Title/Abstract])) OR (Healthcare Provider[Title/Abstract])) OR (Healthcare Provider[MeSH Terms])) OR (Provider, Healthcare[MeSH Terms])) OR (Provider, Healthcare[Title/Abstract])) OR (Healthcare Workers[Title/Abstract])) OR (Healthcare Workers[MeSH Terms])) OR (Healthcare Worker[MeSH Terms])) OR (Healthcare Worker[Title/Abstract])) OR (Health Care Professionals[Title/Abstract])) OR (Health Care Professionals[MeSH Terms])) OR (Health Care Professional[MeSH Terms])) OR (Health Care Professional[Title/Abstract])) OR (Professional, Health Care[Title/Abstract])) OR (Professional, Health Care[MeSH Terms])) AND (Latent Tuberculosis[MeSH Terms])) OR (Latent Tuberculosis[Title/Abstract])) OR (Latent Tuberculoses[Title/Abstract])) OR (Latent Tuberculoses[MeSH Terms])) OR (Tuberculoses, Latent,[MeSH Terms])) OR (Latent Tuberculoses, Tuberculoses, Latent[Title/Abstract])) OR (Tuberculosis, Latent[Title/Abstract])) OR (Tuberculosis, Latent[MeSH Terms])) OR (Latent Tuberculosis[MeSH Terms])) OR (Latent Tuberculosis[Title/Abstract])) OR (Latent Tuberculosis Infection[Title/Abstract])) OR (Latent Tuberculosis Infection[MeSH Terms])) OR (Infection, Latent Tuberculosis[MeSH Terms])) OR (Infection, Latent Tuberculosis[Title/Abstract])) OR (Infections, Latent Tuberculosis[Title/Abstract])) OR (Infections, Latent Tuberculosis[MeSH Terms])) OR (Latent Tuberculosis Infections[MeSH Terms])) OR (Latent Tuberculosis Infections[Title/Abstract])) OR (Tuberculosis Infection, Latent[Title/Abstract])) OR (Tuberculosis Infection, Latent[MeSH Terms])) OR (Tuberculosis Infections, Latent[MeSH Terms])) OR (Tuberculosis Infections, Latent[Title/Abstract])) AND (Occupational Health[Title/Abstract])) OR (Occupational Health[MeSH Terms])) OR (Health, Occupational[MeSH Terms])) OR (Health, Occupational[Title/Abstract])) OR (Industrial Hygiene[Title/Abstract])) OR (Industrial Hygiene[MeSH Terms])) OR (Hygiene, Industrial[MeSH Terms])) OR (Hygiene, Industrial[Title/Abstract])) OR (Industrial Health[Title/Abstract])) OR (Industrial Health[MeSH Terms])) OR (Health, Industrial[MeSH Terms])) OR (Health, Industrial[Title/Abstract])) OR (Safety, Occupational[Title/Abstract])) OR (Safety, Occupational[MeSH Terms])) OR (Occupational Safety[MeSH Terms])) OR (Occupational Safety[Title/Abstract])) OR (Employee Health[Title/Abstract])) OR (Employee Health[MeSH Terms])) OR (Health, Employee[MeSH Terms])) OR (Health, Employee[Title/Abstract])	265

**Figure 1:**
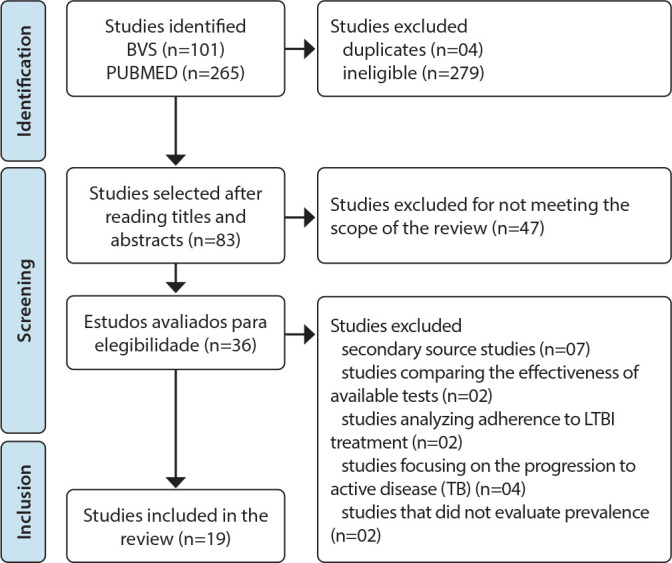
Databases consulted, references retrieved, and selected for the integrative review, 2022

A flowchart was used to organize and select the studies identified in the search, as shown in Figure 1, according to the following stages: identification, screening, and inclusion.

Data extraction was conducted by the principal author, using a recording instrument developed by the review team. The variables included: year, author, title, objectives, study type, country, study location, research participants, databases, diagnostic method for LTBI, prevalence in the study population, and main results related to exposure variables. The data were recorded and organized in an Excel spreadsheet, with the synthesis of review results conducted using descriptive statistics of the diagnostic methods, prevalence, and exposure variables.

## RESULTS

After applying the search key in the referred databases, using the inclusion and exclusion criteria, 366 studies were identified. Of these, 36 were deemed eligible, and after reading the full articles, 19 met the scope of this review (Chart 2).

**Chart 2:** Characterization of the Integrative Review Articles According to Year, Author, Title, Objectives, Study Type, Country, Study Location, and Research Participants (n=19)

Nº	Year, Author, Title	Objectives	Study Type, Country, Location	Research Participants
A1	Prevalence and risk factors for latent tuberculosis infection among primary health care workers in Brazil (Prado et al., 2017)^(7)^.	To determine the prevalence of LTBI and risk factors among healthcare professionals in primary care in five Brazilian cities.	Cross-sectional study conducted in Brazil in a Primary Healthcare Unit.	Primary healthcare professionals - Community health agents, nurses, nursing technicians, and doctors (n=704).
A2	*Infecção por tuberculose entre profissionais de saúde da atenção básica (Lacerda et al., 2017)^(8)^.*	To estimate the prevalence of LTBI and identify associated risk factors among primary healthcare professionals in the municipality of Vitória (ES).	Cross-sectional study conducted in Brazil in a Primary Healthcare Unit.	Primary healthcare professionals (n=218); Community health agents, nursing assistants or technicians, nurses, and doctors.
A3	Occupational Risk of Latent Tuberculosis Infection in Health Workers of 14 Military Hospitals (Yoon et al., 2017)^(9)^.	To determine the prevalence rate of LTBI and occupational risk factors.	Cross-sectional study conducted in South Korea in Military Hospitals.	Healthcare workers (902); (doctors, nurses, laboratory technicians, radiology technicians).
A4	Prevalence and risk factors for latent tuberculosis infection among healthcare workers in Nampula Central Hospital, Mozambique (Belo e Naidoo, 2017)^(10)^.	To evaluate the prevalence and associated risk factors of LTBI among healthcare professionals at the Central Hospital of Nampula, Mozambique.	Cross-sectional study conducted in Mozambique in a Hospital Unit.	Healthcare professionals (209); administrative staff and nurses.
A5	Prevalence and determinants of latent tuberculosis infection among frontline tuberculosis healthcare workers in southeastern China: A multilevel analysis by individuals and health facilities (Chen et al., 2019)^(11)^.	To assess the prevalence of LTBI among healthcare professionals with TB and analyze the factors associated with LTBI at both individual and institutional levels.	Cross-sectional study conducted in China in a Hospital Unit.	Healthcare professionals (487); nursing staff, laboratory technicians, doctors, chest radiologists, administrative staff, and other support personnel.
A6	Prevalence of and factors related to latent tuberculous infection among all employees in a referral hospital (Kim et al., 2018)^(12)^.	To investigate the prevalence and related factors of LTBI among all hospital employees.	Cross-sectional study conducted in South Korea in a Hospital Unit.	Healthcare professionals (479) 36 doctors, 193 nurses, and 250 other professionals.
A7	First assessment of interferon gamma release assay results among healthcare workers at a general hospital in China (Guo et al., 2018)^(13)^.	To evaluate the initial screening of healthcare professionals for LTBI using IGRA in a general hospital in Beijing.	Mixed methods study (Cross-sectional/Longitudinal) conducted in China in a Hospital Unit.	Healthcare professionals (518) Medical staff (doctors and nurses) Non-medical staff (other professionals).
A8	Screening of latent tuberculosis infection among health care workers working in Hajj pilgrimage area in Saudi Arabia, using interferon gamma release assay and tuberculin skin test (Bukhary et al., 2018)^(14)^.	To evaluate the prevalence of LTBI among healthcare professionals working during the Hajj pilgrimage using IGRA and TST and measure their concordance.	Prospective cross-sectional study conducted in Saudi Arabia in a Hospital Unit.	Healthcare professionals (520) - Doctors, nurses, pharmacists, housekeepers, laboratory technicians, radiology technicians.
A9	Difficulties in tuberculosis infection control in a general hospital of Vietnam: a knowledge, attitude, and practice survey and screening for latent tuberculosis infection among health professionals(Ngo et al., 2019)^(15)^.	To clarify the challenges in TB infection control in non-specialized TB hospitals and whether there are associated risks of latent TB infection among healthcare professionals in Vietnam.	Cross-sectional study conducted in Vietnam in a Hospital Unit.	The participants (299) were healthcare professionals, including doctors, nurses, and other professionals.
A10	Evaluation and treatment of latent tuberculosis infection among healthcare workers in Korea: A multicentre cohort analysis (Han et al., 2019)^(16)^.	To describe the testing of healthcare professionals for LTBI and analyze the acceptance and completion of LTBI treatment.	Retrospective cohort study conducted in South Korea in a Hospital Unit.	Healthcare professionals (3920) - doctors; nurses; radiology, laboratory, and pathology technicians; administrative staff; health assistants (professionals who provide physiotherapy services and patient transfers).
A11	Prevalence and risk factors for latent tuberculosis infection among healthcare workers in Morocco (Sabri et al., 2019)^(17)^.	To investigate the prevalence and risk factors for LTBI assessed by IGRAs and Tuberculin Test (TT) among healthcare professionals living in Morocco.	Cross-sectional study conducted in Morocco in a Military Hospital.	Healthcare professionals (662) - The occupation variable was divided into three categories: non-medical (administrative staff, secretaries), paramedical (laboratory technicians and assistants), and medical (doctors and nurses).
A12	Isolation measures and protection awareness are significant for latent tuberculosis infection: a cross-sectional study based on T-SPOT.TB among health care workers in China (Deng et al., 2019)^(18)^.	To reveal the risk factors associated with LTBI detected by the T-SPOT.TB assay among healthcare professionals in different workplaces in China.	Cross-sectional study conducted in China in a Hospital Unit.	Healthcare workers (934) - administrative staff, clinical officers, nurses, doctors, laboratory technicians, patient attendants, ward attendants.
A13	A tuberculin skin test survey among healthcare workers in two public tertiary care hospitals in Bangladesh (Islam et al., 2020)^(19)^.	To determine the prevalence of LTBI and compare the prevalence among healthcare professionals in two public tertiary care hospitals.	Cross-sectional study conducted in Bangladesh in a Hospital Unit.	Hospital healthcare professionals (818 - nurses, doctors, health assistants, laboratory staff).
A14	Risk Analysis of Latent Tuberculosis Infection among Health Workers Compared to Employees in Other Sectors (Hermes et al., 2020)^(20)^.	To assess the additional risk of LTBI among healthcare professionals.	Cross-sectional study conducted in Germany in a Hospital Unit.	The database contains information on 4,882 individuals. Healthcare professionals (doctors, nurses, administrative staff, technicians, interns, and others) and non-healthcare professionals (education, office, chemical industry, service sector (hairdresser), and other professionals in training).
A15	Latent tuberculosis infection among healthcare workers using Quantiferon-TB GoldPlus in a country with a low burden for tuberculosis: prevalence and risk factors (Almohaya et al., 2020)^(21)^.	To determine the prevalence of LTBI in a large heterogeneous population of healthcare professionals and evaluate the risk factors for LTBI.	Cross-sectional and case-control study conducted in Saudi Arabia in a Hospital Unit.	Healthcare professionals 3024 cross-sectional study (294 cases and 294 controls) - doctors, nurses, laboratory technicians, radiology technicians, allied health professions, and non-clinical jobs.
A16	Prevalence of positive TST among healthcare workers in high-burden TB setting in Peru (Sedamano et al., 2020)^(22)^.	To estimate the prevalence of positive Tuberculin Skin Test (TST) and investigate factors associated with a positive TST in healthcare professionals.	Cross-sectional study conducted in Peru in a Primary Care Center.	Healthcare professionals (240) - doctors, nurses, nursing technicians, obstetricians, laboratory technicians, psychologists, nutritionists, social workers, administrative assistants, and other staff.
A17	Decreased annual risk of tuberculosis infection in South Korean healthcare workers using interferon-gamma release assay between 1986 and 2005 (Lee et al., 2021)^(23)^.	To identify the prevalence of LTBI and the annual risk of TB infection among South Korean healthcare professionals based on their interferon-gamma release assays (IGRA).	Retrospective cross-sectional study conducted in South Korea in a Hospital Unit.	Healthcare professionals (3,233) - administrative staff, health assistants (employees who provide physiotherapy services and patient transfers), nurses, doctors, technicians performing laboratory, radiology, and pathology exams.
A18	Risk of latent tuberculosis infection among healthcare workers in Italy: a retrospective study with Quantiferon Test (Coppeta et al., 2021)^(24)^.	To evaluate the prevalence of LTBI among healthcare professionals at the “Tor Vergata” Polyclinic Foundation.	Retrospective study - Registry analysis conducted in Italy in a Polyclinic.	Healthcare professionals (805 - doctors, nurses, and radiology and laboratory technicians).
A19	QuantiFERON-TB Gold plus testing for the detection of LTBI among health care workers in major TB hospitals of the Northern Kyrgyz Republic (Corbett et al., 2022)^(25)^.	To assess the prevalence of LTBI among healthcare professionals in northern Kyrgyzstan and determine the association of LTBI with specific positions or departments.	Cross-sectional study conducted in Kyrgyzstan in a Hospital Unit.	Healthcare workers (404): doctors, nurses, janitors, among others.

The following Chart 3 presents other results of interest found in the articles of the integrative review and is organized as follows: diagnostic method for LTBI, results related to prevalence in the study population, professional category with the highest prevalence of LTBI, and exposure variables:

**Chart 3:** Characterization of the Review according to the diagnostic method for Latent Infection by Mycobacterium tuberculosis, results related to prevalence in the study population, and main results related to exposure variables

Nº	Diagnostic Method for LTBI	Prevalence in the Study Population	Exposure Variables Related to the LTBI Outcome Variable
A1	IGRA - QFT TB Gold	The prevalence of LTBI was 27% (n=196; 95% CI: 24%-31%).	The following factors showed a positive association with LTBI among healthcare professionals in primary care: age > 50 years (OR=2.94; 95% CI: 1.44-5.99), absence of BCG scar (OR=2.10; 95% CI: 1.28-3.43), former smoker (OR=1.80; 95% CI: 1.04-3.11), nurse profession (OR=2.97; 95% CI: 1.13-7.83), nursing technician profession (OR=3.10; 95% CI: 1.26-7.60), community health agent profession (OR=2.60; 95% CI: 1.06-6.40), and irregular use of N95 masks (OR = 2.51; 95% CI: 1.11-5.98).
A2	PPD RT-23 (State Serum Institute,Copenhague, Dinamarca)	The prevalence of positive tuberculin test was 39.4% (95% CI: 32.9%-45.9%) and 54.1% (95% CI: 47.4%-60.7%) for TT cut-off points ≥ 10 mm and ≥ 5 mm, respectively.	Regarding the factors associated with the test result, “having previously done the TST” showed statistically significant associations with a positive TST result for cut-off points ≥ 10 mm and ≥ 5 mm [OR = 2.5 (95% CI: 1.17-5.30) and OR = 2.18 (95% CI: 1.23-3.87), respectively].
A3	PPD RT-23 (State Serum Institute,Copenhague, Dinamarca). **+** IGRA-QTF TB Gold)	The proportion of LTBI in the studied population was 5.8% (52/902).	In the univariate analysis, advanced age, comorbidity, a higher number of BCG scars, and having provided care to TB patients were associated with LTBI. In a multivariate logistic regression analysis, providing care to TB patients for one year or more was the only significant occupational risk factor OR 2.27; 95% confidence interval [CI], 1.13-4.56).
A4	PPD RT-23	The prevalence of LTBI was 34.4% among healthcare professionals.	LTBI was higher among those who had worked for more than eight years (39.3%), those without BCG vaccination (39.6%), and those who were immunocompromised (78.1%). Being immunocompromised was significantly associated with LTBI (OR 5.97[95% CI].
A5	IGRA-TB	33.9% of healthcare professionals tested positive for LTBI.	At the institutional level, a low level of TB epidemic, regular infection control training for healthcare professionals, and regular maintenance of ultraviolet disinfection equipment were significantly associated with a lower rate of LTBI among healthcare professionals. At the individual level, being > 50 years old (OR 2.95 [95% CI 1.43 - 6.07], smoking OR 2.69 [95% CI 1.30 - 5.67], alcohol use OR 2.63 [95% CI 1.38 - 5.00], longer years of work in tuberculosis > 20 years OR 3.41 [95% CI 1.29 - 9.02] and more weekly contact time with TB patients (10 to 40 hours of contact OR 2.15 [95% CI 1.06 - 4.37], > 40 hours of contact, OR 2.88 [95% CI 1.06 - 6.09]) were identified as factors associated with LTBI among the healthcare professionals.
A6	IGRA - QFT GOLD IN TUBE	The overall prevalence of LTBI was 15.7%, 43.1% of whom initiated and completed LTBI treatment.	Compared to healthcare professionals without LTBI, those with LTBI were more likely to be older (P < 0.001), male (P = 0.003), work in low-risk departments (P = 0.013) and have more years of employment (P< 0.001). The prevalence of LTBI was higher in doctors (27.8%), followed by healthcare professionals without patient contact (23.4%), nurses (8.3%) and other healthcare professionals in contact with patients (6.9%). In the multivariate analysis, compared to healthcare professionals <20 years, those aged 40 years had 4.08 times higher odds of having LTBI (P = 0.007). Additionally, compared to healthcare professionals who had worked for <1 year, those who had worked for 1 to 5 years or for >5 years had, respectively, 7.55 (P = 0.014) and 13.69 (P = 0.001) times higher odds of having LTBI.
A7	IGRA - QFT GOLD IN TUBE	The prevalence of LTBI in the target population was 21.8%.	Differences in LTBI prevalence were significantly related to age (P = ), length of employment, and history of exposure to TB at work (OR 1.877 [95% CI 1.170 - 2.711]) for those who perceived no exposure to MTB at work.
A8	PPD RT-23 (State Serum Institute, Copenhagen, Denmark) + IGRA-QFT TB Gold	The rate of LTBI was 10.8% for IGRA and 8.5% for the Tuberculin Skin Test.	The overall agreement between the two tests was poor - 83%. The prevalence of LTBI was associated with a longer length of employment (P = 0.02).
A9	PPD (Not specified)	A high prevalence of latent TB infection (74.2%) was observed, mainly among emergency room participants.	There was higher LTBI positivity in individuals with a record of BCG vaccination, possible contact with TB patients, and professionals working in emergency departments, with a p-value of <0.001.
A10	IGRA - QFT GOLD IN TUBE	There was a prevalence of positive IGRA in 22.8% (893 healthcare professionals out of 3,920).	Overall, 893 healthcare professionals tested positive for IGRA. Among them, 609 healthcare professionals visited the clinic for LTBI evaluation. Of the 609 healthcare professionals evaluated, 302 (49.6%) received treatment for LTBI. The acceptance proportion for treatment was 64.5% (195 of 302 healthcare professionals), and the treatment completion rate was 73.3%.
A11	PPD RT-23 (State Serum Institute, Copenhagen, Denmark) + IGRA - QFT GOLD IN TUBE	The prevalence of IGRA QFT was 40.7% and the positivity of the Tuberculin Sensitivity Test (TST) at the 10mm cut-off point was 52.1%.	A high prevalence of LTBI was observed among professionals in two Moroccan hospitals associated with positive IGRA QFT and TST tests: Male sex (OR 2.14 [95% CI 1.54% - 2.97%], increased age from 35 to 44 years (OR 1.80 [95% CI 1.23% - 2.63%], 45-60 years (OR 4.81 [95% CI 2.72% - 8.52%]), smoking (OR 2.57 [95% CI 1.59% - 4.15%]), family history of TB (OR 3.16 [95% CI 1.63% - 6.12%]), and working in a pulmonology unit (OR 2.31 [95% CI 1.21% - 4.40%]) were consistent risk factors associated with LTBI.
A12	IGRA T-SPOT.TB	The prevalence of LTBI was 28.58% among healthcare professionals.	Healthcare professionals working with hospitalized TB patients (OR 2.91 [95% CI 1.85% - 4.59%] p = < 0.001) and respiratory wards (OR 1.64 [95% CI 1.12% - 3.01%] p = < 0.015) and with longer service in healthcare (OR 1.048 [95% CI 1.016% - 1.080] p = < 0.003) were risk factors for positive T-SPOT (IGRA).
A13	PPD RT-23 (Sachin Gujarat, India)	The prevalence of LTBI among healthcare professionals was 42%.	There was a significant association in healthcare professionals aged 35-45 years (OR 1.36, [95% CI] 1.06-1.73); there was a statistical association in professionals with >3 years of service (OR 1.67, [95% CI] 1.62-1.72) compared to a group with 1.5 years of service; Regarding the workplace, healthcare professionals working in the medical ward (OR 3.65 [95% CI] 2.20-6.05) and in the gynecology and obstetrics ward (OR 2.46 [95% CI] 1.42-4.27) were more likely to have LTBI compared to healthcare professionals working in administrative areas. Females had a higher chance of LTBI (OR 1.08 [95% CI] 1.01 - 1.18) compared to males.
A14	IGRA - QFT GOLD IN TUBE	Healthcare professionals tended to exhibit higher IGRA values than non-healthcare professionals.	Healthcare professionals, when compared to other professionals who are not in the healthcare field, had positive IGRA results with an odds ratio of (OR 3.86 [95% CI] 0.99% - 32.5%; P = 0.05).
A15	IGRA - QFT PLUS	The prevalence of LTBI was 24% in the sample (3024) of healthcare professionals.	A positive QFT-Plus and a diagnosis of LTBI were associated with age over 50 years (OR=1.945 [95% CI] 1.432-2.643), female sex (OR=1.593 [95% CI] 1.310-1.937), nationality (44% Filipinos) - (P≤.001), professional category (P≤.001), and occupation location: in the emergency room (OR=2.415 [95% CI] 1.703-3.425), ICU (OR=2.094 [95% CI] 1.539-2.849).
A16	Tuberculin Skin Test (TST) Tubersol	Overall, 56.5% of participants had a positive TST.	There was a higher prevalence ratio in healthcare professionals working for more than four months in these care centers (PR 1.52 [95% CI] 1.19-1.95, p= 0.001), and for advanced age where a PR of 1.01 and a p-value of 0.004 was obtained.
A17	IGRA QFT-TB GOLD	The positive IGRA rate was 24.6% (LTBI) among healthcare professionals.	The prevalence of LTBI increased with age (6.6% at 20 years, 34.3% at 40 years, and about 50% at 60 years, respectively (OR 13.3 [95% CI] 7.02-25.3 p= <0.001)); healed tuberculosis lesion on chest radiography (OR 6.24 [95% CI] 3.26-11.9 p= <0.001), male sex (OR 2.29 [95% CI] 1.93-2.73 p= <0.001), and length of employment: from 240 to <360 months (OR 6.11 [95% CI] 4.15-9.01 p= 0.001) and >360 months (OR 8.61 [95% CI] 5.50-13.48 p= 0.001) when compared to less than 12 months of employment were risk factors for positivity.
A18	IGRA QFT-TB GOLD	The prevalence of LTBI was 4.5% of the sample.	The study highlights the low prevalence of LTBI in Italian healthcare workers. There was a significant statistical association for the variable sex with a higher frequency of LTBI (p= 0.015).
A19	IGRA QFT-TB GOLD	There was a prevalence of LTBI of 46.7% found among the professionals in the study.	There was an increased likelihood of having a positive QFT test for: the position of doctor (OR 8.7 [95% CI] 1.2-60.5 p= 0.03) and laboratory staff (OR 19.8 [95% CI] 2.9-135.4 p<0.01) when the administration staff was used as the baseline. When comparing departments across all hospitals, laboratories (OR 7.65 [95% CI] 2.3-24.9 p<0.001), negative TB smear (OR 5.90 [95% CI] 1.6-21.8 p= 0.008), surgery (OR 3.79 [95% CI] 1.3-11.4 p= 0.018), and outpatient clinic (OR 3.80 [95% CI] 1.1-13.0 p= 0.03) had a higher odds ratio for positive QFT tests when the administrative department was used as the baseline.

*Notes: IGRA QFT - TB: Interferon Gamma Release Assay - QuantiFERON-TB Gold In-Tube test. PPD: Purified Protein Derivative, RT: Reset Tuberculin. LTBI: Latent Tuberculosis Infection.*

## DISCUSSION

Since this study presents a review of the scientific literature on the prevalence of LTBI and its association with possible risk factors among healthcare workers, the Asian continent represented the majority of these scientific productions (63.15%), followed by South America (15.78%), Europe, and Africa, both with a representation of 10.52% of the productions.

In a geographical context, in 2020, 43% of TB cases occurred in the World Health Organization (WHO) Southeast Asia regions, 25% in Africa, 3.0% in the Americas, followed by Europe with 2.3% of cases. From this perspective, the regions of the African and Asian continents together represented 68% of TB cases in 2020, which may be related to the higher quantity of productions found in this review, where the two continents together account for 73.67% ^(26)^.

Furthermore, of the 12 countries in which the scientific productions were carried out, 66.6% (Bangladesh, Vietnam, Brazil, Peru, South Korea, Mozambique, Kyrgyzstan, and China) are present in at least one of the three global lists of countries with a high burden of TB, TB associated with HIV, and Multidrug-Resistant and Rifampicin-Resistant Tuberculosis (MDR/RR-TB), with China being present on all three lists ^(26)^.

Of the total studies, 78.9% took place in hospitals ^(9-21,23,25)^, while the other studies used locations such as primary healthcare units and polyclinics ^(7,8,22,24)^. The study populations involved healthcare workers with variations among professional categories. Nursing was the only category that participated in all studies. Several authors point to healthcare professionals as a risk group for LTBI and invest in understanding the risk factors associated with infection ^(7-25)^.

In this review, the prevalence varied between 4.5% (lowest value found) and 56.8% (highest value found); the lowest prevalence was in a study conducted in Italy ^(24)^, which is a country considered by WHO to have a low incidence of TB ^(27)^; the highest prevalence was found in a study conducted in Vietnam ^(15)^, which is a country considered by WHO as one of the 30 countries with a high burden of TB and MDR/RR-TB ^(26)^.

A similar result to the highest prevalence of LTBI in healthcare professionals found in this review (56.8%) was in a study conducted in Peru, which aimed to estimate the prevalence of LTBI in healthcare professionals. This study identified a prevalence rate of 56.5% ^(22)^. It is worth noting that both studies (Vietnam and Peru) used the tuberculin skin test for the diagnosis of LTBI, considering a reading value of 5mm induration for a positive result.

It is noteworthy that Peru, although not on the list of countries with a high TB burden (unlike Vietnam), is considered a country with a high incidence of MDR/RR-TB among its cases ^(28)^.

Still considering the context above, Prado et al. ^(7)^, in a study aiming to determine the prevalence of LTBI and associated risk factors among healthcare professionals in primary care, found a higher odds ratio among the categories of nursing technicians (OR 3.10 [95% CI] 1.26-7.60), community health agents (OR 2.60 [95% CI] 1.06-6.40), and nurses (OR 2.97 [95% CI] 1.13-7.83) compared to the professional category of doctors.

Contrasting with the previous study, Kim et al. ^(12)^, considering a different setting (a hospital in South Korea), showed in their analysis that working as a doctor, compared to other professions, represented a higher odds ratio (OR 5.19 [95% CI] 1.49-18.13) for exposure to LTBI.

Two other studies found similar statistical results regarding the higher exposure of the medical category to LTBI: One study conducted in hospitals in Saudi Arabia showed a higher prevalence (20%) with a p-value of 0.02 for positive IGRA results in doctors compared to other professions ^(14)^, and another study conducted in hospitals in the Republic of Kyrgyzstan found a rate of positive interferon-gamma release assays (IGRA-QFT) in doctors of 65.2% and an OR of 8.7 [95% CI] 1.2-60.5^(25)^.

Another study conducted in a hospital in Saudi Arabia showed a higher exposure to LTBI for professionals in clinical areas compared to non-clinical areas; however, the following professional categories represented higher exposure: nurses (OR 2.67 [95% CI] 2.11-3.37), health assistants (OR 2.08 [95% CI] 1.49-2.89), and radiology technicians showing the highest exposure (OR 3.08 [95% CI] 1.58-6.03) ^(21)^. In contrast, Lee et al. ^(23)^, in a study conducted in a hospital in South Korea, found in their analysis that being a nurse in the studied setting might be a protective factor compared to other categories (OR 0.2 [95% CI] 0.15-0.25).

Thus, it should be considered that environments where people with active pulmonary or laryngeal disease are present and expelling aerosols through coughing, talking, or sneezing pose some risk of transmission ^(29)^.

It is emphasized that prevention measures, biosafety, the provision and use of PPE, and health worker surveillance actions become important for strengthening actions aimed at reducing MTB infection in healthcare services, thus preventing the onset of active disease and its transmissibility ^(30)^.

Regarding the diagnosis of LTBI, it can be performed using the tuberculin skin test (TST) or interferon-gamma release assays (IGRAs). Unlike the tuberculin test, IGRAs are high-cost tests capable of quantifying the synthesis of interferon-gamma produced by T lymphocytes (CD4+ and CD8+) due to sensitization to specific MTB antigens. As they do not present cross-reactions with the BCG vaccine and other non-tuberculous mycobacteria species, they are more specific compared to the PPD ^(31,32)^.

In this review, for the diagnosis of LTBI, most studies (57.89%) used some type of interferon-gamma release assay (IGRA) ^(7,11-13,16,18,20,21,23-25)^, while 26.31% of the studies ^(8,10,15,19,22)^ used only the PPD as the diagnostic method for LTBI, and only 15.78% used both diagnostic methods (IGRAs and PPD) ^(9,14,17)^.

Of the studies that used PPD as a diagnostic method for LTBI, 50% used PPD RT-23 from the State Serum Institute, Copenhagen, Denmark ^(8,9,14,17)^. Still in this context, regarding the reading and positivity of the tuberculin tests used in the studies of this review, the authors considered the following values: for PPD reading: 37.5% of the authors ^(8,9,17)^ considered a time of 72 hours after inoculation; in one study (15), it was not possible to find information about the reading time. The other studies, which account for 50% of those that used the tuberculin test as a diagnostic method for LTBI, considered a shorter reading time for PPD (48 hours) ^(10,14,19,22)^.

In Brazil, there is a consensus for the reading of the tuberculin test, which can vary between 48 to 96 hours after inoculation, but the best inflammatory response is observed within 72 hours ^(29)^. Regarding induration, some authors considered a positive test in healthcare professionals with a cut-off point greater than or equal to 10mm, and for immunocompromised professionals, a value equal to or greater than 5mm ^(8,9,22)^. Other authors used higher cut-off points (equal to or greater than 15mm) considering individuals vaccinated with BCG ^(15)^.

In Brazil, a test is considered positive if an individual obtains a value equal to or greater than 5mm in a tuberculin test, regardless of their vaccination status or immunological condition ^(3)^. Knowing that some skin tests (PPD) can have cross-reactions with the BCG vaccine ^(3)^, it becomes relevant to discuss the statistically significant results found in studies that use vaccination history or the presence of a vaccination scar as a variable with possible associations such as the time since vaccination and false-positive results. In this regard, some authors considered higher cut-off points for induration to minimize false-positive results.

Thus, Yoon et al. ^(9)^, in their study, associated a higher prevalence in healthcare professionals with a greater number of BCG scars (p < 0.001); considering the high immunization rate in the country (South Korea), they established a higher cut-off point for induration (10 mm) to consider the tuberculin test positive to minimize false-positive results.

Using the same line of reasoning, Ngo et al. ^(15)^, in their research conducted in a hospital in Vietnam, found in their analysis a relationship between the prevalence of positive tuberculin tests (49.1%) in healthcare professionals and a history of BCG vaccination; to reduce possible bias, they considered an even higher cut-off point than the study by Yoon et al. ^(9)^, thus for healthcare professionals vaccinated with BCG (without other considerations), a positive test was considered to be ≥ 15 mm.

It is important to note that neither study reported the timing of BCG vaccination, which can influence the skin test results, as ten years after BCG vaccination, only 1% of positive tuberculin tests can be attributed to BCG ^(29)^.

Regarding exposure variables, apart from those already discussed, the categories analyzed in all the studies (sex and age) were chosen. Those with a significant association with the LTBI outcome variable, both for tuberculin tests and IGRAs, were considered for discussion.

In terms of sex, an association with LTBI was found in the study by YOON et al. ^(9)^, where females had a higher prevalence than males (33.3% vs. 24.9%) with a p-value of 0.014 considering the tuberculin test. There was no statistical relationship with IGRA; however, in the study by Bukhary et al. ^(14)^, an association with LTBI was found in the IGRA test, with females being more prevalent than males, 94.2% and 86.8%, respectively (p = 0.01). Conversely, Coppeta et al. ^(24)^ showed in their analysis that males were related to LTBI with twice the prevalence compared to females (p = 0.01).

Sabri et al. ^(17)^ also found in their analysis a higher odds ratio for a positive IGRA test in males (OR 2.14 [95% CI] 1.54 - 2.97) compared to females. Regarding age and considering the positivity relationship in both tuberculin tests and IGRAs, the studies showed a trend for LTBI in older age groups ^(7,9,11,12,14,17-25)^.

## Study Limitations

The inclusion criterion used was full-text articles only, which might have resulted in the exclusion of other relevant studies. Another limitation was the time frame, possibly leading to the omission of recent studies. Additionally, the exclusion of other databases in this review and the fact that data extraction was not performed independently by two reviewers also represent limitations. From an epidemiological perspective, it is noteworthy that the segmentation of the age variable into various age groups posed a significant challenge to the analysis.

## Contributions to the Field

In light of the above, this review contributes to a better understanding of latent MTB infection among healthcare workers, thereby facilitating the adoption of new surveillance strategies aimed at reducing MTB infection in healthcare services. In the same vein, the importance of early diagnosis of LTBI and preventive TB treatment among healthcare workers is highlighted. The research aligns with the guiding pillars for combating the disease in the country, whose objectives are to reduce the incidence and mortality rates of the disease in Brazil.

## FINAL CONSIDERATIONS

In this review, due to the chosen time frame, the reduced number of studies found in Brazil were conducted only in primary care, which hinders the understanding of LTBI in the country’s hospital healthcare services. The plurality of healthcare teams and the structure of healthcare services in different countries make it difficult to compare the results of these studies.

The studies pointed to a significant association between being a healthcare professional and being exposed to LTBI, with a potentially higher risk for professionals working in countries with a higher incidence rate of active disease. This review also showed that being a doctor or nurse and being older, in general, are related to TB infection.

LTBI in this review was more related to males compared to females. Another issue is that despite the high cost of interferon-gamma release assays, this review showed a preference for these tests compared to the tuberculin skin test.

Additionally, the different tests used for the diagnosis of LTBI and their varying cut-off points for positivity (in the case of skin tests) may have influenced the prevalence results found. Regarding tuberculin skin tests, it is noted that some authors increase the cut-off points for induration at the time of reading to reduce potential biases. This highlights the need for more studies on the implementation of skin tests to evaluate cross-reactions with the BCG vaccine, especially in countries with high vaccination rates.

Regarding skin tests, studies that use the presence of a BCG vaccination scar as a variable should include the timing of the vaccination to facilitate deeper discussions about the relationship between skin test positivity and the vaccine.

Based on the results of the studies included in this review, more studies on the epidemiology of LTBI, particularly in the context of healthcare workers, are necessary to contribute to a better understanding and to formulate more impactful actions that protect healthcare workers. Effective biosafety actions can consequently reduce the risk of illness, helping to mitigate the incidence and prevalence indicators of TB worldwide.
