# Supplementary material for: Prevalence and exposure variables of latent infection by mycobacterium tuberculosis in healthcare workers
Source: Rev Bras Enferm. 2025 Jan 10;77(Suppl 2):e20240052. doi: 10.1590/0034-7167-2024-0052 (PMC11726814; doi:10.1590/0034-7167-2024-0052)
Supplement: Translation

# Translation: Prevalência e variáveis de exposição da Infecção Latente pelo *Mycobacterium tuberculosis* em Trabalhadores de Saúde

**DOI:** 10.1590/0034-7167-2024-0052pt

**Authors:** Raphael Sampaio dos Santos, Katerine Moraes dos Santos, Angela Maria Mendes Abreu, Cristiane Cardoso de Paula, Regina Célia Gollner Zeitoune

**Affiliations:** I Universidade Federal do Rio de Janeiro. Rio de Janeiro, Rio de Janeiro, Brasil; II Universidade Federal Fluminense. Rio de Janeiro, Rio de Janeiro, Brasil; III Universidade Federal de Santa Maria. Santa Maria, Rio Grande do Sul, Brasil

**Keywords:** Pessoal da Saúde, Tuberculose Latente, Saúde do Trabalhador, Tuberculose, Prevalência

## Abstract

**Objetivos:**

Identificar na literatura científica a prevalência, os meios de diagnósticos e variáveis de exposição da Infecção Latente pelo *Mycobacterium Tuberculosis* em trabalhadores da saúde.

**Métodos:**

Revisão integrativa da literatura científica a partir da seguinte pergunta de revisão: Quais as evidências científicas disponíveis na literatura que abordam a prevalência da infecção latente pelo Mycobacterium Tuberculosis em trabalhadores da saúde e sua associação com possíveis fatores de risco entre estes trabalhadores?

**Resultados:**

Ser médico ou enfermeiro, ter idade mais avançada e ser do sexo masculino, em geral, está associado a maiores prevalências. O estudo mostrou também que, os ensaios de liberação de interferon gama, foram mais utilizados como meio diagnóstico quando comparados aos testes cutâneos.

**Considerações Finais:**

São necessários mais estudos em relação à epidemiologia da Infecção Latente pelo Mycobacterium Tuberculosis no contexto dos trabalhadores da saúde visando ações de maior impacto contribuindo para redução da Tuberculose no mundo.

## INTRODUÇÃO

A tuberculose (TB) permanece como um importante problema de saúde pública, e estima-se que um quarto da população global já tenha se infectado pelo *Mycobacterium tuberculosis* (MTB). Além disso, a doença tem um grande impacto econômico nos serviços de saúde dos países, com consequências para as famílias afetadas, que enfrentam custos catastróficos (custos totais anuais sofridos pelas famílias, relacionados direta e indiretamente à TB, superiores a 20% de sua renda) ^(1)^.

Um estudo realizado no Brasil, que teve como objetivo avaliar o impacto econômico domiciliar do adoecimento por TB, revelou que 41% dos pacientes participantes com TB experimentaram custos catastróficos, tendo como fatores determinantes ser paciente e chefe da família, viver na pobreza antes da TB, emprego informal atual e parar de trabalhar durante a doença^(2)^. Nesse sentido, têm-se intensificado os esforços para eliminar a TB no mundo^(1)^.

No contexto da TB, destaca-se a Infecção Latente pelo *Mycobacterium tuberculosis* (ILTB), que se caracteriza por um estado de resposta imune persistente ao bacilo sem evidência de doença ativa, mas com grande importância do ponto de vista epidemiológico, já que uma pessoa infectada pelo MTB pode desenvolver a doença e transmiti-la para outros indivíduos saudáveis, sustentando assim a cadeia de transmissão da doença^(3)^.

O Brasil, entre outros países, vem se empenhando para atingir os Objetivos do Desenvolvimento Sustentável (ODS), estabelecidos pela Agenda 2030, legitimada no ano de 2015. Sendo assim, faz parte do escopo desta revisão integrativa a discussão da ILTB em profissionais de saúde, destacando o ODS 3, que tem como objetivo assegurar a saúde e o bem-estar para todos, sendo uma das metas eliminar a TB e outras epidemias até 2030 ^(4)^.

Os profissionais de saúde, devido à exposição frequente, fazem parte da população de risco para adquirir TB e suas cepas resistentes. O risco de transmissão da doença pode variar de uma unidade de saúde para outra, sobretudo no ambiente hospitalar, e até mesmo em diferentes lugares dentro da mesma unidade, de acordo com a maior ou menor circulação de doentes ^(5)^. Em virtude disso, e tendo em vista a vulnerabilidade dos profissionais de saúde à ILTB, o risco de progressão para a doença ativa e o entendimento do seu papel na cadeia de transmissão da TB nos serviços de saúde, faz-se necessário entender o rastreamento da infecção latente pelo MTB entre esses trabalhadores.

Considerando a eliminação da TB até 2030 como uma das metas do desenvolvimento sustentável e como uma política mundial de saúde ^(4)^, e tendo em vista a relevância de entender o papel do profissional da saúde na cadeia de transmissão da doença, a presente pesquisa buscou identificar as evidências científicas disponíveis na literatura que abordam a infecção latente pelo MTB em trabalhadores da saúde e sua associação com possíveis fatores de risco.

## OBJETIVO

Identificar na literatura científica estudos que versam sobre a prevalência da ILTB em trabalhadores da saúde e possíveis variáveis de exposição à infecção por TB.

## MÉTODOS

### Aspectos éticos

Trata-se de um estudo que utilizou dados de domínio público e não envolveu seres humanos, portanto, não houve necessidade de apreciação pelo Comitê de Ética em Pesquisa.

### Desenho do estudo

Foi realizada uma revisão integrativa seguindo os procedimentos metodológicos: Definição da pergunta de revisão, que auxilia na delimitação do tópico de interesse, utilizando, nesta revisão, o acrônimo PICo, referente aos elementos estruturantes População, Interesse e Contexto; Busca e seleção de estudos primários, a partir do estabelecimento dos critérios de inclusão e exclusão dos estudos, busca destes em bases de dados, organização do banco de referências e seleção dos estudos; Extração de dados dos estudos, utilizando um instrumento de registro e organização dos dados extraídos dos estudos selecionados; Avaliação crítica dos estudos a partir dos resultados apresentados e nível de evidência; Síntese dos resultados da revisão e discussão de suas evidências, identificação das lacunas do conhecimento sobre o tópico de interesse, limitações da revisão e recomendações; Apresentação da revisão ^(6)^.

### Período e local do estudo

A busca foi realizada no mês de maio de 2022 na base de dados Centro Latino-Americano e do Caribe de Informações em Ciências da Saúde, acessada por meio da Biblioteca Virtual em Saúde - BVS (BIREME), e no *Medical Literature Analysis and Retrieval System Online* (MEDLINE), acessado por meio da *National Library of Medicine* (PubMed).

### Critérios de inclusão e exclusão

Foram critérios de inclusão artigos completos diretamente na base de dados nos idiomas português, inglês, espanhol ou francês, que abordaram a prevalência da infecção latente pelo MTB, desenvolvidos em serviços de saúde hospitalares, ambulatoriais e de atenção primária à saúde, cuja população foi composta por trabalhadores de saúde, independente da área de formação e nível de escolaridade. Foi estabelecido como recorte temporal os últimos 05 (cinco) anos, esta escolha se deve à publicação, em 2018, do Protocolo de Vigilância da Infecção Latente pelo MTB no Brasil pelo então Departamento de Vigilância das Doenças Transmissíveis, cujo objetivo principal é oferecer aos profissionais de saúde e aos programas de controle da TB subsídios para a implantação, ampliação e fortalecimento da vigilância da ILTB em seus territórios, caracterizando-se como um marco político nacional na temática. Foram excluídos da análise manuais, diretrizes, dissertações, teses, guias, editoriais e comentários.

### Protocolo do estudo

Foi elaborada a seguinte pergunta de revisão: “Quais as evidências científicas disponíveis na literatura que abordam a prevalência da infecção latente pelo MTB em trabalhadores da saúde e sua associação com possíveis fatores de risco entre estes trabalhadores?”. Conforme o acrônimo PICo, a população compreendeu os trabalhadores da saúde, o fenômeno de interesse, a infecção latente pelo MTB e o contexto, os serviços de saúde, porém, ao inserir os descritores relacionados, os resultados foram muito inespecíficos, dessa forma optou-se por utilizar a saúde do trabalhador como contexto.

Para auxílio nesta etapa foi construído o Mapa Conceitual da estratégia PICo, utilizando o vocabulário controlado dos Descritores em Ciências da Saúde (DECS) e *Medical Subject Headings* (MESH), incluindo os termos alternativos e *entry terms*. A partir do vocabulário controlado foram construídas chaves de busca utilizando-se os operadores booleanos OR para integração e AND para diferenciação entre os termos, conforme apresentado no Quadro 1. Ainda na PubMed, aplicou-se o filtro de idade (acima de 19 anos), por se tratar de trabalhadores de saúde.

**Quadro 1:** Chaves de busca utilizadas nas bases de dados e quantidade de resultados encontrados

Base de dados	Chave P+I+Co	Resultado
Biblioteca virtual em saúde (BVS) *Utilizando* termos alternativos	(Pessoal da Saúde) OR (Prestadores de Cuidados de Saúde) OR (Profissionais da Saúde ) OR (Profissionais de Saúde ) OR (Profissional da Saúde ) OR (Profissional de Saúde ) OR (Trabalhador da Saúde ) OR (Trabalhador de Saúde ) OR (Trabalhadores da Saúde ) OR (Trabalhadores de Saúde) AND (Tuberculose Latente) OR (Infecção Tuberculosa Latente) AND (Saúde do Trabalhador) OR (Higiene do Trabalho) OR (Higiene Industrial) OR (Saúde dos Empregados) OR (Saúde dos Trabalhadores) OR (Saúde Industrial) OR (Saúde Ocupacional) OR (Segurança do Trabalho) OR (Segurança dos Trabalhadores) OR (Segurança no Trabalho) OR (Segurança Ocupacional)	101
*National Library of Medicine (Pubmed)* Utilizando os *entry terms*	*(((((((((((((((((((((((((((((((((((((((((((((((((((((((((((((((((((Health Personnel[Title/Abstract]) OR (Health Personnel[MeSH Terms])) OR (personnel, health[MeSH Terms])) OR (Personnel, Health[Title/Abstract])) OR (Health Care Providers[Title/Abstract])) OR (Health Care Providers[MeSH Terms])) OR (Health Care Provider[MeSH Terms])) OR (Health Care Provider[Title/Abstract])) OR (Provider, Health Care[Title/Abstract])) OR (Provider, Health Care[MeSH Terms])) OR (Healthcare Providers[MeSH Terms])) OR (Healthcare Providers[Title/Abstract])) OR (Healthcare Provider[Title/Abstract])) OR (Healthcare Provider[MeSH Terms])) OR (Provider, Healthcare[MeSH Terms])) OR (Provider, Healthcare[Title/Abstract])) OR (Healthcare Workers[Title/Abstract])) OR (Healthcare Workers[MeSH Terms])) OR (Healthcare Worker[MeSH Terms])) OR (Healthcare Worker[Title/Abstract])) OR (Health Care Professionals[Title/Abstract])) OR (Health Care Professionals[MeSH Terms])) OR (Health Care Professional[MeSH Terms])) OR (Health Care Professional[Title/Abstract])) OR (Professional, Health Care[Title/Abstract])) OR (Professional, Health Care[MeSH Terms])) AND (Latent Tuberculosis[MeSH Terms])) OR (Latent Tuberculosis[Title/Abstract])) OR (Latent Tuberculoses[Title/Abstract])) OR (Latent Tuberculoses[MeSH Terms])) OR (Tuberculoses, Latent,[MeSH Terms])) OR (Latent Tuberculoses, Tuberculoses, Latent[Title/Abstract])) OR (Tuberculosis, Latent[Title/Abstract])) OR (Tuberculosis, Latent[MeSH Terms])) OR (Latent Tuberculosis[MeSH Terms])) OR (Latent Tuberculosis[Title/Abstract])) OR (Latent Tuberculosis Infection[Title/Abstract])) OR (Latent Tuberculosis Infection[MeSH Terms])) OR (Infection, Latent Tuberculosis[MeSH Terms])) OR (Infection, Latent Tuberculosis[Title/Abstract])) OR (Infections, Latent Tuberculosis[Title/Abstract])) OR (Infections, Latent Tuberculosis[MeSH Terms])) OR (Latent Tuberculosis Infections[MeSH Terms])) OR (Latent Tuberculosis Infections[Title/Abstract])) OR (Tuberculosis Infection, Latent[Title/Abstract])) OR (Tuberculosis Infection, Latent[MeSH Terms])) OR (Tuberculosis Infections, Latent[MeSH Terms])) OR (Tuberculosis Infections, Latent[Title/Abstract])) AND (Occupational Health[Title/Abstract])) OR (Occupational Health[MeSH Terms])) OR (Health, Occupational[MeSH Terms])) OR (Health, Occupational[Title/Abstract])) OR (Industrial Hygiene[Title/Abstract])) OR (Industrial Hygiene[MeSH Terms])) OR (Hygiene, Industrial[MeSH Terms])) OR (Hygiene, Industrial[Title/Abstract])) OR (Industrial Health[Title/Abstract])) OR (Industrial Health[MeSH Terms])) OR (Health, Industrial[MeSH Terms])) OR (Health, Industrial[Title/Abstract])) OR (Safety, Occupational[Title/Abstract])) OR (Safety, Occupational[MeSH Terms])) OR (Occupational Safety[MeSH Terms])) OR (Occupational Safety[Title/Abstract])) OR (Employee Health[Title/Abstract])) OR (Employee Health[MeSH Terms])) OR (Health, Employee[MeSH Terms])) OR (Health, Employee[Title/Abstract])*	265

Para a organização e seleção dos estudos identificados na busca utilizou-se a um fluxograma, conforme explicitado na Figura 1, de acordo com as etapas: identificação, triagem e inclusão.

**Figura 1:** Fluxograma da busca: Bases de dados consultadas, referências recuperadas e selecionadas para a revisão integrativa, 2022

A extração de dados foi desenvolvida pelo autor principal, com a utilização de um instrumento de registro elaborado pela equipe de revisão, que constou das variáveis: ano, autor, título, objetivos, tipo de estudo, país, local do estudo, participantes da pesquisa, bases de dados, meio de diagnóstico para ILTB, prevalência na população do estudo e principais resultados relacionados às variáveis de exposição. O registro e organização dos dados foi desenvolvido em uma planilha Excel, sendo a síntese dos resultados da revisão conduzida por estatística descritiva dos meios de diagnóstico, a prevalência e variáveis de exposição.

## RESULTADOS

Após a aplicação da chave de busca nas referidas bases de dados, utilizando os critérios de inclusão e exclusão, foram identificados 366 estudos, sendo elegíveis 36 e após a leitura dos artigos na íntegra, 19 atenderam ao escopo desta revisão (Quadro 2).

**Quadro 2:** Caracterização dos artigos da Revisão Integrativa de acordo com ano, autor, título, objetivos, tipo de estudo, país, local do estudo e participantes da pesquisa (n=19)

Nº	Título, autor e ano	Objetivos	Tipo de estudo, país e local	Participantes da pesquisa
A1	*Prevalence and risk factors for latent tuberculosis infection among primary health care workers in Brazil (Prado et al., 2017)^(7)^.*	Determinar a prevalência da ILTB e fatores de risco entre profissionais de saúde na atenção primária em cinco cidades brasileiras.	Estudo Transversal, realizado no Brasil em Unidade Básica de Saúde.	Profissionais de saúde atenção primária - Agentes Comunitários de saúde, enfermeiros, técnicos de enfermagem e médicos (n= 704).
A2	Infecção por tuberculose entre profissionais de saúde da atenção básica (Lacerda et al., 2017)^(8)^.	Estimar a prevalência de ILTB e identificar os fatores de risco associados a essa infecção entre profissionais de saúde da atenção básica no município de Vitória (ES).	Estudo Transversal, realizado no Brasil em Unidade Básica de Saúde.	Profissionais de saúde de atenção primária (n=218); Agentes comunitários de saúde, auxiliares ou técnicos de enfermagem, enfermeiros e médicos.
A3	*Occupational Risk of Latent Tuberculosis Infection in Health Workers of 14 Military Hospitals (Yoon et al., 2017)^(9)^.*	Determinar a taxa de prevalência de ILTB, bem como fatores de risco ocupacionais.	Estudo Transversal realizado na Coreia do Sul em Hospitais Militares.	Trabalhadores da área de saúde (902); (médicos, enfermeiros, técnicos em laboratório, técnico radiológico).
A4	*Prevalence and risk factors for latent tuberculosis infection among healthcare workers in Nampula Central Hospital, Mozambique (Belo e Naidoo, 2017)^(10)^.*	Avaliar a prevalência e os fatores de risco associados à ILTB entre os profissionais de saúde do Hospital Central de Nampula, Moçambique.	Estudo Transversal, realizado em Moçambique em unidade Hospitalar.	Profissionais da saúde (209); funcionários administrativos e enfermeiros.
A5	*Prevalence and determinants of latent tuberculosis infection among frontline tuberculosis healthcare workers in southeastern China: A multilevel analysis by individuals and health facilities (Chen et al., 2019)^(11)^.*	Avaliar a prevalência de ILTB entre os profissionais de saúde com TB e analisar os fatores associados à ILTB em nível individual e institucional.	Estudo Transversal realizado na China em unidade Hospitalar.	Profissionais de saúde (487); Profissionais da equipe de enfermagem, técnico em laboratório, médicos, radiologista de tórax, pessoal administrativos e outros de apoio.
A6	*Prevalence of and factors related to latent tuberculous infection among all employees in a referral hospital (Kim et al., 2018)^(12)^.*	Investigar a prevalência e os fatores relacionados à ILTB entre todos os funcionários do hospital.	Estudo transversal realizado na Coreia do Sul em unidade Hospitalar.	Profissionais de saúde (479) 36 médicos, 193 enfermeiros e 250 outros profissionais).
A7	*First assessment of interferon gamma release assay results among healthcare workers at a general hospital in China (Guo et al., 2018)^(13)^.*	Avaliar a primeira triagem de profissionais de saúde para ILTB usando o IGRA em um hospital geral em Pequim.	Estudo de métodos mistos Transversal/Longitudinal realizado na China em em unidade Hospitalar.	Profissionais de saúde (518) Equipe médica (médicos e enfermeiros) Equipe não médica (outros profissionais)
A8	*Screening of latent tuberculosis infection among health care workers working in Hajj pilgrimage area in Saudi Arabia, using interferon gamma release assay and tuberculin skin test (Bukhary et al., 2018)^(14)^.*	Avaliar a prevalência de ILTB entre profissionais de saúde que trabalham na peregrinação do Hajj usando IGRA e TST e medir sua concordância.	Estudo transversal prospectivo realizado na Arábia Saudita em unidade Hospitalar.	Profissionais de saúde 520 (Médicos, Enfermeiros, farmacêutico, Governanta, Técnico de laboratório, Técnico de radiologia).
A9	*Difficulties in tuberculosis infection control in a general hospital of Vietnam: a knowledge, attitude, and practice survey and screening for latent tuberculosis infection among health professionals(Ngo et al., 2019)^(15)^.*	Esclarecer as dificuldades no controle da infecção por TB em hospitais não especializados em TB e se existem riscos associados de infecção latente por TB entre os profissionais de saúde no Vietnã	Estudo Transversal realizado no Vietnã em em unidade Hospitalar.	Os participantes (299) foram profissionais de saúde, incluindo médicos, enfermeiros e outros profissionais.
A10	*Evaluation and treatment of latent tuberculosis infection among healthcare workers in Korea: A multicentre cohort analysis (Han et al., 2019)^(16)^.*	Descrever o teste de profissionais de saúde para ILTB e analisar a aceitação e conclusão do tratamento de ILTB.	Estudo de coorte retrospectivo, realizado na Coreia do Sul em em unidade Hospitalar.	Profissionais de saúde (3920)- médicos; enfermeiros; técnicos em radiologia, laboratório e patologia; administrativos; auxiliares de saúde ( profissionais que realizam serviços de fisioterapia e transferências de pacientes).
A11	*Prevalence and risk factors for latent tuberculosis infection among healthcare workers in Morocco (Sabri et al., 2019)^(17)^.*	Investigar a prevalência e os fatores de risco para ILTB avaliados por IGRAs e Teste Tuberculínico (TT) em profissionais de saúde que vivem no Marrocos.	Estudo Transversal realizado no Marrocos em Hospital Militar.	Profissionais de saúde (662) A variável ocupação foi dividida em três categorias: não médicos (administrativos, secretários), para médicos ( técnicos e auxiliares de laboratório) e médica (médicos e enfermeiros)
A12	*Isolation measures and protection awareness are significant for latent tuberculosis infection: a cross-sectional study based on T-SPOT.TB among health care workers in China (Deng et al., 2019)^(18)^.*	Revelar os fatores de risco associados para ILTB detectados pelo ensaio T-SPOT-TB entre profissionais de saúde em diferentes locais de trabalho na China.	Estudo transversal realizado na China em unidade Hospitalar.	Trabalhadores de saúde (934 - pessoal administrativo, oficiais clínicos, enfermeiras, médicos; técnico de laboratório; atendentes de pacientes; atendentes de ala;
A13	*A tuberculin skin test survey among healthcare workers in two public tertiary care hospitals in Bangladesh (Islam et al., 2020)^(19)^.*	Determinar a prevalência de ILTB e comparar a prevalência entre profissionais de saúde em dois hospitais públicos de atenção terciária.	Estudo Transversal realizado em Bangladesh em unidade Hospitalar.	Profissionais de Saúde hospitalar (818- enfermeiros, médicos, auxiliares de saúde; pessoal de laboratório).
A14	*Risk Analysis of Latent Tuberculosis Infection among Health Workers Compared to Employees in Other Sectors (Hermes et al., 2020)^(20)^.*	Avaliar o risco adicional de profissionais de saúde para ILTB.	Estudo Transversal realizado na Alemanha em unidade Hospitalar.	O banco de dados possui informações de 4.882 pessoas. Profissionais de saúde (médicos, enfermeiros, administrativos, técnicos, estagiários, e outros) e não profissionais de saúde (educação, escritório, indústria química, setor de serviço (cabeleireiro) e outros profissionais em formação;
A15	*Latent tuberculosis infection among healthcare workers using Quantiferon-TB GoldPlus in a country with a low burden for tuberculosis: prevalence and risk factors (Almohaya et al., 2020)^(21)^.*	Determinar a prevalência de ILTB em uma grande população heterogênea de profissionais de saúde e avaliar os fatores de risco para ILTB.	Estudo transversal e de caso-controle realizado na Arábia Saudita em unidade Hospitalar.	Profissionais de Saúde 3024 estudo transversal (294 casos e 294 controle) - médicos enfermeiros, técnicos de laboratório, técnico de radiologia, profissões afins à saúde e empregos não clínicos;
A16	*Prevalence of positive TST among healthcare workers in high-burden TB setting in Peru (Sedamano et al., 2020)^(22)^.*	Estimar a prevalência de *Tuberculin Skin Test* (TST) positivo e para investigar fatores associados a um TST positivo em profissionais de saúde.	Estudo transversal realizado no Peru em Centro de cuidados primários.	Profissionais de saúde (240) médicos, enfermeiros, técnicos em enfermagem, obstetras, técnico de laboratório, psicólogos, nutricionistas, assistente social, assistente administrativo e outra equipe.
A17	*Decreased annual risk of tuberculosis infection in South Korean healthcare workers using interferon-gamma release assay between 1986 and 2005 (Lee et al., 2021)^(23)^.*	Identificar a prevalência de ILTB e Risco anual de infecção por TB entre profissionais de saúde sul-coreanos com base em seus ensaios de liberação de interferon-gama (IGRA).	Estudo transversal retrospectivo realizado na Coreia do Sul em unidade hospitalar.	Profissionais de saúde (3.233) administrativos, auxiliares à saúde (funcionários que prestam serviços de fisioterapia e transferência de pacientes), enfermeiros, médicos , técnicos que realizam exames laboratoriais, radiológicos e de patologia.
A18	*Risk of latent tuberculosis infection among healthcare workers in Italy: a retrospective study with Quantiferon Test (Coppeta et al., 2021)^(24)^.*	Avaliar a prevalência de ILTB entre os profissionais de saúde da Fundação Policlínica “Tor Vergata”.	Estudo Retrospectivo - Análise de registros realizado na Itália em Policlínica.	Profissionais de saúde (805 - médicos, enfermeiros e técnicos de radiologia e laboratório).
A19	*QuantiFERON-TB Gold plus testing for the detection of LTBI among health care workers in major TB hospitals of the Northern Kyrgyz Republic (Corbett et al., 2022)^(25)^.*	Avaliar a prevalência de ILTB entre profissionais de saúde no norte da República do Quirguizistão e determinar a associação de ILTB com cargos ou departamentos.	Estudo Transversal realizado na República do Quirguizistão em unidade Hospitalar	Trabalhador da saúde (404): médicos, enfermeiros, faxineiros, entre outros.

A seguir, o Quadro 3 apresenta outros resultados de interesse encontrados nos artigos da revisão integrativa e está organizado da seguinte forma: meio de diagnóstico para ILTB, resultados relacionados à prevalência na população do estudo, categoria profissional de maior prevalência para ILTB e variáveis de exposição:

**Quadro 3:** Caracterização dos artigos da Revisão Integrativa de acordo com o meio de diagnóstico para Infecção Latente pelo *Mycobacterium tuberculosis*, resultados relacionados à prevalência na população do estudo e principais resultados relacionados às variáveis de exposição

Nº	Meio de diagnóstico para ILTB	Prevalência na população do estudo	Variáveis de exposição em relação a variável desfecho ILTB
A1	IGRA - QFT TB Gold	A prevalência de ILTB foi de 27% (n=196; IC95%: 24%-31%).	Os seguintes fatores mostraram associação positiva com ILTB entre profissionais de saúde na atenção primária: idade > 50 anos (OR=2,94; IC95%: 1,44-5,99), ausência de cicatriz de BCG (OR=2,10; IC95%: 1,28-3,43), ex-tabagista (OR=1,80; IC95%: 1,04-3,11), profissão enfermeiro (OR=2,97; IC95%: 1,13-7,83), profissão técnico de enfermagem (OR=3,10; IC95%: 1,26-7,60), profissão agente comunitário de saúde (OR=2,60; IC95%: 1,06-6,40) e uso irregular de máscaras N95 (OR = 2,51; IC95%: 1,11-5,98).
A2	PPD RT-23 (State Serum Institute,Copenhague, Dinamarca)	A prevalência de positividade ao teste tuberculínico foi de 39,4% (IC95%: 32,9-45,9) e de 54,1% (IC95%: 47,4-60,7), respectivamente, para os pontos de corte do TT ≥ 10 mm e ≥ 5 mm	Em relação aos fatores associados ao resultado do teste, “ter feito TT anteriormente” apresentou associações estatisticamente significativas com o resultado positivo ao TT para os pontos de corte ≥ 10 mm e ≥ 5 mm [OR = 2,5 (IC95%: 1,17-5,30) e OR = 2,18 (IC95%: 1,23-3,87), respectivamente].
A3	PPD RT-23 (State Serum Institute,Copenhague, Dinamarca). **+** IGRA-QTF TB Gold)	A proporção de ILTB na população estudada foi de 5,8% (52/902).	Na análise univariada, idade avançada, comorbidade, maior número de cicatrizes de BCG e ter prestado assistência ao paciente de TB foram associados à ILTB. Em uma análise de regressão logística multivariada, fornecer assistência ao paciente com TB por um ano ou mais foi o único fator de risco ocupacional significativo OR 2,27; intervalo de confiança de 95% [IC], 1,13-4,56).
A4	PPD RT-23	A prevalência de ILTB foi de 34,4% entre os profissionais da saúde.	A ILTB foi maior naqueles que trabalhavam há mais de oito anos (39,3%), naqueles que não tinham vacina BCG (39,6%) e eram imunocomprometidos (78,1%). Ser imunocomprometido foi associado significativamente à ILTB (OR 5,97[IC 95%].
A5	IGRA-TB	33,9% dos profissionais de saúde testaram positivo para ILTB	No nível institucional, um baixo nível de epidemia de TB, treinamento regular de controle de infecção para profissionais de saúde e manutenção regular de equipamentos de desinfecção ultravioleta foram significativamente associados a uma menor taxa de ILTB entre profissionais de saúde. No nível individual, ter > de 50 anos (OR 2,95 [IC95% 1,43 - 6,07], fumar OR 2,69 [IC95% 1,30 - 5,67], o uso de álcool OR 2,63 [IC95% 1,38 - 5,00], maior número de anos de trabalho em tuberculose > 20 anos OR 3,41 [IC95% 1,29 - 9,02 e maior tempo semanal de contato com pacientes com tuberculose (de 10 à 40 horas de contato OR 2,15 [ IC95% 1,06 - 4,37], > 40 horas de contato, OR 2,88 [ IC95% 1,06 - 6,09]) foram identificados como fatores associados para ILTB entre os profissionais de saúde.
A6	IGRA - QFT GOLD IN TUBE	A prevalência geral de ILTB foi de 15,7%, 43,1% dos quais iniciaram e completaram o tratamento de ILTB	Comparando os profissionais de saúde sem ILTB, aqueles com ILTB tinham maior probabilidade de serem mais velhos (*P* < 0,001), do sexo masculino (*P* = 0,003), trabalhar em departamentos de baixo risco ( *P* = 0,013) e ter mais anos de emprego (*P*< 0,001). A prevalência de ILTB foi maior em médicos (27,8%), seguido por profissionais de saúde sem contato com o paciente (23,4%), enfermeiros (8,3%) e outros profissionais de saúde em contato com pacientes (6,9%). Na análise multivariada, em comparação com os profissionais de saúde <20 anos, aqueles com idade 40 anos tiveram 4,08 vezes mais chances de ter ILTB (*P* = 0,007). Além disso, em comparação com profissionais de saúde que trabalham há <1 ano, aqueles que trabalham por 1 a 5 anos ou por 5 anos tiveram, respectivamente, 7,55 ( *P* = 0,014) e 13,69 ( *P* = 0,001) vezes mais chances de ter ILTB.
A7	IGRA - QFT GOLD IN TUBE	A prevalência de ILTB na população-alvo foi de 21,8%.	Diferenças na prevalência de ILTB foram significativamente relacionadas à idade (*P* = , tempo de trabalho e histórico de exposição à TB no trabalho (OR 1,877 [IC95% 1,170 - 2,711] ) para aqueles que perceberam não ter exposição ao MTB no trabalho.
A8	PPD RT-23 (State Serum Institute,Copenhague, Dinamarca) **+** IGRA-QFT TB Gold)	A taxa de ILTB foi de 10,8% para o IGRA e 8,5% para o Teste tuberculínico.	A concordância geral entre os dois testes foi ruim - 83%. A prevalência de ILTB foi associada a um tempo de emprego mais longo (*P* = 0,02).
A9	PPD (Não informado)	Observou-se alta prevalência de infecção latente por TB (74,2%), principalmente entre os participantes do pronto-socorro.	Houve uma maior positividade para ILTB em indivíduos com registro de vacina BCG, possível contato com paciente TB e em profissionais que trabalhavam em pronto socorro, sendo o valor de *p=<0,001*.
A10	IGRA - QFT GOLD IN TUBE	Houve uma prevalência de IGRA positivo em 22.8% (893 profissionais de saúde de 3.920).	No geral, 893 profissionais de saúde foram positivos para IGRA. Entre eles, 609 profissionais de saúde visitaram a clínica para avaliação de ILTB. Dos 609 profissionais de saúde avaliados, 302 (49,6%) receberam tratamento para ILTB. A proporção de aceitação para tratamento foi de 64,5% (195 dos 302 profissionais de saúde), sendo a taxa de conclusão do tratamento de 73,3%.
A11	PPD RT-23 (State Serum Institute,Copenhague, Dinamarca) + IGRA - QFT GOLD IN TUBE	Houve prevalência de IGRA QFT de 40,7% e de positividade do teste de sensibilidade tuberculínico (TST) no ponto de corte de 10mm foi de 52,1%.	Foi observada uma elevada prevalência de ILTB entre os profissionais em dois hospitais marroquinos associados ao teste IGRA QFT positivo e ao TST positivo: Sexo masculino (OR 2,14 [IC95% 1,54%- 2,97%], aumento da idade de 35 à 44 anos (OR 1,80 [IC95% 1,23%- 2,63%],45-60 anos (OR 4,81 [IC95% 2,72%- 8,52%], tabagismo (OR 2,57 [IC95% 1,59%- 4,15%], historia familiar de TB (OR 3,16 [IC95% 1,63%- 6,12%] e trabalho em unidade de pneumologia (OR 2,31 [IC95% 1,21%- 4,40%] foram fatores de risco consistentes associados ao ILTB.
A12	IGRA T-SPOT.TB	Houve prevalência de ILTB em 28,58% dos profissionais de saúde.	Profissionais de saúde que trabalham com pacientes internados com TB (TB) (OR 2,91 [IC 95% 1,85%- 4,59%] *p=* < 0,001) e enfermarias respiratórias (OR 1,64 [IC 95% 1,12%- 3,01%] *p=* < 0,015) e com maior tempo de serviço de saúde (OR 1,048 [IC 95% 1,016% - 1,080] *p=* < 0,003) foram fatores de risco para T-SPOT (IGRA) positivo.
A13	PPD RT-23 (Sachin Gujarat, Índia)	A prevalência de ILTB entre os profissionais de saúde foi de 42%.	Houve associação significativa nos profissionais de saúde com idade entre 35-45 anos (OR 1,36, [IC 95%] 1,06-1,73); houve associação estatística em profissionais com > 3 anos de serviço (OR 1,67, [IC 95%] 1,62-1,72) quando comparados a um grupo com 1,5 anos de serviço; Em relação ao local de trabalho, profissionais de saúde que atuam na enfermaria médica (OR 3,65 [IC 95%] 2,20-6,05), e na enfermaria de ginecologia e obstetrícia (OR 2,46 [IC 95%] 1,42-4,27) tiveram maior chance de ILTB em comparação aos profissionais de saúde que trabalhavam em áreas administrativas. Pessoas do sexo feminino tiveram uma maior chance para ILTB, (OR 1,08 [IC 95%] 1,01 - 1,18) quando comparadas ao sexo masculino.
A14	IGRA - QFT GOLD IN TUBE	Os Profissionais de saúde tendiam a exibir valores IGRA mais elevados do que os nãoprofissionais de Saúde.	Profissionais de saúde quando comparados a outros profissionais que não são da área de saúde, tiveram resultados positivos para IGRA com uma razão de chance correspondente à (OR 3,86 [IC95%] 0,99% - 32,5%; *P* = 0,05).
A15	IGRA - QFT PLUS	A prevalência de ILTB foi de 24% da amostra (3024) dos profissionais de saúde.	Um QFT-Plus positivo e um diagnóstico de ILTB foram associados a idade superior a 50 anos (OR=1,945 [IC 95%] 1,432-2,643), sexo feminino (OR=1,593 [IC 95%] 1,310-1,937), nacionalidade (44% Filipinos) - (P≤,001), categoria profissional (P≤.001), e local de ocupação: em pronto-socorro (OR=2.415 [IC 95%] 1,703-3,425), UTI (OR=2,094 [ IC 95%] 1,539-2.849). Nas análises de caso controle, houve associação estatística significativa para: faixa etária acima dos 50 anos (OR = 2,79, [IC 95%] 1,49-5,23), para exposição conhecida à TB (OR=1,9, [IC 95%] 1,37-2,65), e por não receber BCG ao nascimento (OR = 3,13, [IC95%] 1,22-8,05).
A16	Teste Tuberculínico (TST) Tubersol.	No geral, 56,5% dos participantes tiveram um TT positivo.	Houve uma razão de prevalência maior em profissionais de saúde que trabalham por mais de quatro meses nestes centros de cuidados (PR 1,52 [IC 95%] 1,19-1,95, *p=* 0,001, e para idade avançada onde se obteve uma PR 1,01 e o valor de *p=0,004.*
A17	IGRA QFT-TB GOLD	A taxa positiva de IGRA foi de 24,6% (ILTB) nos profissionais de saúde.	A prevalência de ILTB aumentou conforme o avanço da idade (6,6% aos 20 anos e 34,3% aos 40 anos e cerca de 50% aos 60 anos respectivamente (OR 13,3 [95%] 7,02-25,3 *p=* <0,001); lesão de Tuberculose cicatrizada na radiografia de tórax (OR 6,24 [95%] 3,26-11,9 *p=* <0,001), sexo masculino (OR 2,29 [95%] 1,93-2,73 *p=* <0,001,) e tempo de trabalho sendo: de 240 à <360 meses (OR 6,11 [95%] 4,15 - 9,01 *p*=0,001) e >360 (OR 8,61 [95%] 5,50 - 13,48 *p*=0,001) quando comparados a um tempo inferior a 12 meses de trabalho. meses foram fatores de risco para positividade de
A18	IGRA QFT-TB GOLD	A prevalência de ILTB foi de 4,5% da amostra.	O estudo destaca a baixa prevalência do ILTB em trabalhadores italianos na área da saúde. Houve associação estatística significativa para variável sexo à maior frequência de ILTB ( *p*= 0,015).
A19	IGRA QFT-TB GOLD PLUS	Houve uma prevalência de ILTB de 46,7% encontrado nos profissionais do estudo.	Houve um aumento das probabilidades de ter um teste QFT positivo para: a posição de médico (OR 8,7 [95%] 1,2 - 60,5 *p=0,03*) equipe de laboratório (OR 19,8 [95%] 2,9 - 135,4 *p<0,01*) quando a equipe de administração foi utilizada como o linha de base. Ao comparar os departamentos de todos os hospitais juntos, laboratórios (OR 7,65 [IC95%] 2,3-24,9 *p*<0,001), esfregaço negativo de TB (OR 5,90 [IC95%] 1,6-21,8 *p*=0,008), cirurgia (OR 3,79 [IC95%] 1,3-11,4 *p*=0,018), e clínica ambulatorial (OR 3,80 [IC95%] 1,1-13,0 *p*=0,03) tiveram uma maior razão de chance para testes de QFT positivo quando utilizado como linha de base o departamento administrativo.

*Notas: IGRA QFT - TB: Interferon Gamma Release Assay - QuantiFERON-TB Gold In-Tube test. PPD - Purified Protein Derivatite. ILTB: Infecção latente por tuberculose.*

## DISCUSSÃO

Tratando-se de estudo que apresenta revisão da literatura científica sobre a prevalência de ILTB e sua associação com possíveis fatores de risco em trabalhadores da saúde, o continente asiático, representou o total destas produções científicas (63,15%), seguido pela América do Sul (15,78%), Europa e África, ambos com uma representação de 10,52% das produções.

Em um contexto geográfico, em 2020, 43% dos casos de TB ocorreram nas regiões da Organização Mundial de Saúde (OMS) do Sudeste Asiático, 25% na África, 3,0% nas Américas, seguido da Europa com 2,3% dos casos. Nesta perspectiva, as regiões dos continentes da África e Ásia, em 2020, representaram juntas 68% dos casos de TB, o que pode ter uma relação com um maior quantitativo das produções encontradas nesta revisão, onde os dois continentes somam juntos 73,67%^(26)^.

Além disto, dos 12 países nos quais as produções científicas foram realizadas, 66,6% (Bangladesh, Vietnã, Brasil, Peru, Coréia do Sul, Moçambique, Quirguistão e China) estão presentes em pelo menos uma das três listas globais de países com alta carga de TB, TB associada ao HIV e Tuberculose Multirresistente e Tuberculose Resistente à rifampicina (MDR/RR-TB), estando a China presentes em todas as três listas^(26)^.

Do total dos estudos, 78,9% ocorreram no hospital^(9-21,23,25)^, enquanto os outros estudos utilizaram locais como unidade básica de saúde e policlínicas^(7,8,22,24)^. A população dos estudos envolveu trabalhadores da área da saúde com variação entres as categorias profissionais. A enfermagem foi a única categoria participante em todos os estudos. Diversos autores apontam os profissionais de saúde como grupo de risco para o ILTB e investem em entender os fatores de risco associados à infecção^(7-25)^.

Nesta revisão, a prevalência variou entre 4,5% (menor valor encontrado) e 56,8% (maior valor encontrado); a menor prevalência, foi em um estudo que ocorreu na Itália^(24)^, que é um país considerado pela OMS de baixa incidência de TB^(27)^; a maior prevalência encontrada, ocorreu em um estudo realizado no Vietnã^(15)^, que é um país considerado pela OMS um dos 30 países com uma alta carga de TB e MDR/RR-TB^(26)^.

Resultado similar a maior prevalência de ILTB em profissionais de saúde encontrada nesta revisão (56,8%), foi em um estudo realizado no Peru, que tinha como objetivo estimar a prevalência da ILTB em profissionais de saúde. Neste estudo, foi identificada uma taxa de prevalência de 56,5%^(22)^. Cabe destacar, que ambos os estudos (Vietnã e Peru), utilizaram o teste tuberculínico para o diagnóstico da ILTB, considerando um valor de leitura de 5mm na enduração para um resultado positivo.

Chama atenção, o Peru, que embora não esteja na lista de países com alta carga de TB (ao contrário do Vietnã), é um país considerado com alta incidência de MDR/RR-TB entre seus casos^(28)^.

Ainda considerando o contexto acima, Prado et al^(7)^, em um estudo com objetivo de determinar a prevalência de ILTB e fatores de risco associados em profissionais de saúde na atenção primária, encontrou uma maior razão de chance entre as categorias de técnico de enfermagem (OR 3,10 [IC 95%] 1.26-7.60, agentes comunitários de saúde (OR 2.60 [95%] 1.06 - 6.40) e enfermeiros (OR 2.97 [IC 95%] 1.13-7.83) quando comparados a categoria profissional de médico.

Contrastando com o estudo anterior Kim et al^(12)^ considerando um cenário diferente (hospital da Coréia do Sul), mostrou em sua análise que trabalhar como médico, comparado a outras profissões, representou uma maior razão chance (OR 5.19 [IC95%] 1.49 - 18.13) para exposição à ILTB.

Outros dois estudos, encontraram resultados estatísticos semelhantes em relação a maior exposição da categoria médica em relação a exposição à ILTB: 01 estudo realizado em hospitais na Arábia Saudita, mostrou uma maior prevalência (20%) com valor de *p* = 0,02 para resultados positivos em IGRA em médicos quando comparado a outras profissões^(14)^, e o outro estudo, ocorreu em hospitais na República do Quirguizistão e encontrou uma taxa de ensaios de liberação de interferon-gama (IGRA-QFT) positivo em médicos de 65,2% e uma OR 8,7 [95%] 1,2 - 60,5^(25)^.

Outra pesquisa que ocorreu em um hospital na Arábia Saudita, mostrou uma maior exposição à ILTB para profissionais de áreas clínicas, quando comparados a áreas não clínicas; contudo as seguintes categorias profissionais representaram maior exposição: enfermeiros (OR 2,67 [95%] 2.11 - 3.37), auxiliar de saúde (OR 2,08 [95%] 1.49 - 2.89) e técnico em radiologia apresentando uma maior exposição (OR 3,08 [95%] 1.58-6.03)^(21)^; em contraponto ao exposto Lee et al^(23)^ em um estudo realizado em um hospital na Coreia do Sul, encontrou em sua análise que ser enfermeiro no cenário estudado pode ser um fator de proteção relacionado a outras categorias (OR 0,2 [95%] 0,15 - 0,25).

Sendo assim, devemos considerar que ambientes onde circulam pessoas com a doença ativa nas formas pulmonar ou laríngea e que estão eliminando aerossóis através de tosse, fala ou espirro, oferecem algum risco de transmissão^(29)^.

Ressalta-se que, medidas de prevenção, biossegurança, oferta e utilização dos EPI, e as ações de vigilância em saúde do trabalhador, tornam-se importantes para o fortalecimento de ações que visem reduzir a infecção pelo MTB nos serviços de saúde, prevenindo assim o adoecimento para doença ativa e sua transmissibilidade^(30)^.

Quanto ao diagnóstico da ILTB, este pode ser realizado por meio do teste tuberculínico (testes cutâneos) ou por ensaios de liberação de interferon-gama (IGRAs). Os IGRAs, diferentemente da prova tuberculínica, são exames de alto custo, e são capazes de quantificar a síntese do interferon gama produzidos por linfócitos T (CD4+ e CD8+) devido a sensibilização a determinados antígenos específicos do MTB. Por não apresentarem reação cruzada com a vacina BCG, e outras espécies de micobactérias não tuberculosas, são mais específicos quando comparados ao PPD^(31,32)^.

Nesta revisão, para o diagnóstico de ILTB, a maior parte dos estudos (57,89%), utilizaram algum tipo de ensaio de liberação de interferon gama (IGRA)^(7,11-13,16,18,20,21,23-25)^, enquanto 26,31% dos estudos^(8,10,15,19,22)^, utilizaram somente o PPD como meio diagnóstico da ILTB e apenas 15,78%, utilizaram os dois meios de diagnósticos (IGRAs e PPD)^(9,14,17)^.

Dos estudos que utilizaram o PPD como meio diagnóstico para ILTB, (50%) utilizaram o PPD RT-23 State Serum Institute, Copenhagen, Dinamarca^(8,9,14,17)^. Ainda neste contexto, em relação à leitura e a positividade dos testes tuberculínicos utilizados nos estudos desta revisão, os autores consideraram os seguintes valores: em relação a leitura do PPD: 37,5% dos autores^(8,9,17)^ consideraram um tempo após 72 horas da inoculação; em 01 estudo^(15)^, não foi possível encontrar a informação sobre o tempo de leitura. Os outros estudos que correspondem à 50% do total dos que utilizaram o teste tuberculínico como meio diagnóstico para a ILTB, consideraram um tempo menor para leitura do PPD (48 horas)^(10,14,19,22)^.

No Brasil, existe um consenso para a leitura da prova tuberculínica, podendo variar entre 48 a 96 horas após a inoculação, porém a melhor resposta inflamatória é percebida no período de 72 horas^(29)^. Em relação a enduração, alguns autores consideraram para um teste positivo nos profissionais de saúde, um ponto de corte maior ou igual à 10mm sendo para profissionais imunocomprometidos, valor igual ou maior que 5 mm^(8,9,22)^. Outros autores utilizaram ponto de corte maiores (igual ou maior que 15mm) levando em consideração indivíduos que foram vacinados com BCG^(15)^.

No Brasil, considera-se um teste positivo o indivíduo que obtiver em um teste tuberculínico valor igual ou maior que 5 mm independentemente da sua situação vacinal ou estado imunológico^(3)^. Sabendo que alguns testes cutâneos (PPD) podem sofrer reação cruzada com a vacina BCG^(3)^, torna-se relevante discutir os resultados estatisticamente significativos encontrados em pesquisas que utilizam como variável a história de vacinação ou presença de cicatriz vacinal com possíveis associações como o tempo de vacinação e resultados falso positivos. Neste sentido, alguns autores, consideraram pontos de corte maiores na enduração a fim de minimizar resultados falsos-positivos.

Desta forma, Yoon et al^(9)^, em seu estudo, associou uma maior prevalência em profissionais de saúde com maior número de cicatrizes de BCG (*p= < 0,001)*; considerando a alta taxa de imunização no país (Coreia do Sul), a fim de minimizar os resultados falso-positivos nos resultados do teste tuberculínico utilizado, estabeleceram um ponto de corte maior na enduração (10 mm de corte) para considerar a positividade do teste.

Usando a mesma linha de raciocínio Ngo et al^(15)^, em sua pesquisa, que ocorreu em um hospital no Vietnã, encontraram em sua análise uma relação na prevalência nos profissionais de saúde com teste tuberculínicos positivos (49,1%), com registro da vacina da BCG no passado; pensando em reduzir um possível viés, considerou um ponto de corte ainda maior que no estudo de Yoon et al^(9)^, tendo assim para profissionais de saúde vacinados com BCG (sem outras considerações), um teste positivo, ≥ 15mm.

É importante ressaltar que nenhum dos dois estudos relataram o tempo em que a vacinação da BCG foi realizada, o que pode influenciar no resultado do teste cutâneo, já que dez anos após a BCG, apenas 1% das provas tuberculínicas positivas, pode ser atribuída à BCG^(29)^.

Quanto às variáveis de exposição, com exceção das que já foram discutidas anteriormente, foram escolhidas as que foram categorias de análise em todos os estudos (sexo e idade). Foram consideradas para discussão, as que tiveram associação significativa com a variável de desfecho ILTB, tanto para testes tuberculínicos como para IGRAs.

Tendo em vista a variável sexo encontrou-se associação com a ILTB, no estudo de YOON et al^(9)^, onde o sexo feminino teve uma prevalência maior em relação ao masculino (33,3% VS 24,9%) com valor de p= 0,014 considerando o teste tuberculínico. Não houve relação estatística com IGRA; já no estudo de Bukhary et al^(14)^, foi achada associação à ILTB no teste IGRA, sendo o sexo feminino mais prevalente que o sexo masculino 94,2% e 86,8% respectivamente (*p=0,01);* ao contrário, Coppeta et al^(24)^, mostrou em sua análise que o sexo masculino está relacionado à ILTB com o dobro da prevalência se comparado ao sexo feminino ( *p=0,01).* Sabri et al^(17)^, também em sua análise encontrou uma maior razão de chance no teste IGRA positivo no sexo masculino OR 2,14 [95%] 1,54 - 2,97 comparado ao sexo feminino.

Em relação a idade e considerando a relação de positividade tanto em testes tuberculínicos quanto em IGRAs, os estudos mostraram uma tendência para a ILTB nos grupos com mais idade^(7,9,11,12,14,17-25)^.

### Limitações do Estudo

Foi utilizado como critério de inclusão apenas artigos na íntegra, podendo haver perda de outros estudos pertinentes. Uma outra limitação, foi atribuída ao recorte temporal, podendo haver perda de estudos recentes. Além dessas limitações mencionadas, soma-se a não inclusão de outras bases de dados nesta revisão e a extração de dados não ter sido realizada de modo duplo independente. Enfim, em uma perspectiva epidemiológica, destaca-se que nos estudos abordados nesta revisão, a segmentação da variável idade em diversas faixas etárias impõe um desafio significativo à sua análise.

### Contribuições para a Área

Em face do exposto, esta revisão colabora com o maior entendimento da infecção latente pelo MTB entre trabalhadores da área da saúde, permitindo assim a adoção de novas estratégias de vigilância com desdobramento para a redução da infecção pelo MTB nos serviços de saúde. Neste mesmo raciocínio, destacam-se as ações de diagnóstico precoce da ILTB e do tratamento preventivo da TB entre trabalhadores da saúde. A pesquisa também vai ao encontro dos pilares norteadores para o enfrentamento da doença no País, cujos objetivos são a redução dos coeficientes de incidência e mortalidade da doença no Brasil.

## CONSIDERAÇÕES FINAIS

Nesta revisão, devido ao marco temporal escolhido, o número reduzido de estudos encontrados no Brasil nesta revisão, foram conduzidos apenas na atenção primária, o que dificulta o entendimento da ILTB nos serviços de saúde hospitalar do País. Em decorrência da pluralidade das equipes de saúde e da constituição dos serviços de saúde nos diferentes países, dificulta a comparabilidade entre os resultados destes estudos.

Os estudos apontaram para uma associação significativa em ser profissional da área da saúde e estar exposto à ILTB, podendo estar relacionado a um maior risco, os profissionais que trabalham em países de maior coeficiente de incidência da doença ativa. Esta revisão, mostrou também que ser médico ou enfermeiro, ter mais idade, em geral, está relacionado à TB infecção.

A ILTB nesta revisão, esteve mais relacionada ao sexo masculino quando comparada ao feminino. Uma outra questão, é que apesar do custo alto dos ensaios de liberação de interferon gama, esta revisão mostrou que há uma predileção nestes testes quando comparados ao teste tuberculínico.

Adicionalmente, é que os diferentes testes utilizados para o diagnóstico da ILTB e seus diferentes pontos de corte para positividade (quando se tratou dos testes cutâneos), podem ter influenciado nos resultados encontrados de prevalência.

Em relação aos testes tuberculínicos, percebe-se que alguns autores, para reduzir possíveis vieses, aumentam os pontos de corte na enduração no momento da leitura. Isto nos faz pensar na necessidade de mais estudos sobre a realização de testes cutâneos com objetivo de avaliar reações cruzadas com a vacina BCG, principalmente em países onde a taxa de vacinação é alta.

Tratando-se ainda dos testes cutâneos, é necessário que os estudos que utilizam a presença de cicatriz da vacina BCG como variável incluam o tempo em que a vacina foi realizada a fim de abrir discussões mais profundas quanto a relação da positividade do teste cutâneo com a vacina.

Face aos resultados dos estudos que compuseram esta revisão, se faz necessário mais estudos em relação à epidemiologia da ILTB, principalmente no contexto dos trabalhadores da saúde, a fim de contribuir para seu melhor entendimento e para formular ações de maior impacto que contribuam para proteção da população de trabalhadores da saúde, com ações de biossegurança efetivas, consequentemente reduzindo o risco de adoecimento contribuindo assim para atenuar os indicadores de incidência e prevalência da TB no mundo.
